# Towards Biologically-Inspired Visual SLAM in Dynamic Environments: IPL-SLAM with Instance Segmentation and Point-Line Feature Fusion

**DOI:** 10.3390/biomimetics10090558

**Published:** 2025-08-22

**Authors:** Jian Liu, Donghao Yao, Na Liu, Ye Yuan

**Affiliations:** Institute of Machine Intelligence, University of Shanghai for Science and Technology, Shanghai 200093, China; liujian92@usst.edu.cn (J.L.); 223332432@st.usst.edu.cn (D.Y.); liuna@usst.edu.cn (N.L.)

**Keywords:** biologically-inspired perception, dynamic environments, instance segmentation, point-line feature fusion, semantic point cloud, visual SLAM

## Abstract

Simultaneous Localization and Mapping (SLAM) is a fundamental technique in mobile robotics, enabling autonomous navigation and environmental reconstruction. However, dynamic elements in real-world scenes—such as walking pedestrians, moving vehicles, and swinging doors—often degrade SLAM performance by introducing unreliable features that cause localization errors. In this paper, we define dynamic regions as areas in the scene containing moving objects, and dynamic features as the visual features extracted from these regions that may adversely affect localization accuracy. Inspired by biological perception strategies that integrate semantic awareness and geometric cues, we propose Instance-level Point-Line SLAM (IPL-SLAM), a robust visual SLAM framework for dynamic environments. The system employs YOLOv8-based instance segmentation to detect potential dynamic regions and construct semantic priors, while simultaneously extracting point and line features using Oriented FAST (Features from Accelerated Segment Test) and Rotated BRIEF (Binary Robust Independent Elementary Features), collectively known as ORB, and Line Segment Detector (LSD) algorithms. Motion consistency checks and angular deviation analysis are applied to filter dynamic features, and pose optimization is conducted using an adaptive-weight error function. A static semantic point cloud map is further constructed to enhance scene understanding. Experimental results on the TUM RGB-D dataset demonstrate that IPL-SLAM significantly outperforms existing dynamic SLAM systems—including DS-SLAM and ORB-SLAM2—in terms of trajectory accuracy and robustness in complex indoor environments.

## 1. Introduction

Simultaneous Localization and Mapping (SLAM) is a fundamental research area in robotics, aiming to enable autonomous agents to localize themselves and build maps of unknown environments simultaneously. Over the past decade, numerous open-source SLAM systems have been developed, including Cartographer [1], Gmapping [2], Hector SLAM [3], and Karto SLAM [4], most of which are based on laser sensors. In contrast, Visual SLAM (VSLAM) is favored for its cost effectiveness and rich scene information, making it widely applicable in fields such as autonomous driving, augmented reality, virtual reality, and industrial automation. However, in dynamic environments the presence of moving objects and occlusions significantly increases the complexity of data association and degrades localization accuracy.

Several mature visual SLAM systems have been proposed, such as ORB-SLAM2 [5], LSD-SLAM [6], RGB-D SLAM [7], DSO [8], and SVO [9]. Although these methods have made notable progress, most of them rely on the assumption of a static environment [10,11], ignoring or not adequately handling dynamic elements. In real-world scenarios, moving pedestrians, vehicles, and animals are commonly present within the robot’s perception range, posing considerable challenges to SLAM systems.

To address this issue, two major classes of solutions have emerged. Geometric approaches [12,13] filter dynamic features using optical flow or epipolar constraints, but often fail in texture-poor scenes or when multiple moving objects exist. In contrast, semantic-based methods—such as Dyna-SLAM [14] and DS-SLAM [15]—leverage deep neural networks to detect and remove dynamic regions. While effective in many cases, they are sensitive to segmentation errors, especially at object boundaries, and their accuracy degrades when dynamic content dominates the scene. Furthermore, traditional point-only representations lack sufficient geometric constraints in low-feature-density environments, motivating the integration of richer structural cues such as line features.

**Figure 1:**
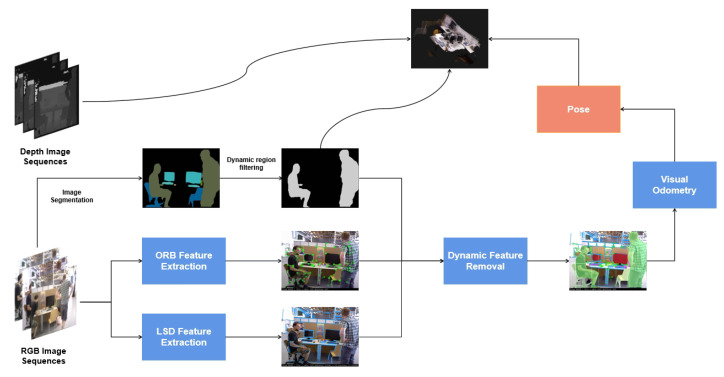
Modular pipeline of the proposed IPL-SLAM system.

Motivated by the way biological visual systems integrate semantic awareness and geometric structure for robust perception in dynamic surroundings, we propose a novel visual SLAM framework called IPL-SLAM. Our method fuses instance-level segmentation with point and line feature extraction to achieve enhanced robustness and accuracy. An overview of the system is shown in Figure 1. Built upon ORB-SLAM2 [5], IPL-SLAM adds parallel threads for YOLOv8-based segmentation and point cloud map construction. We extract ORB and LSD features, perform motion consistency checks and angle analysis to remove dynamic points and lines, and employ an adaptive-weight error function for pose optimization. The system ultimately produces a static semantic point cloud map for high-level scene understanding. Experiments on dynamic sequences from the TUM RGB-D dataset validate that IPL-SLAM achieves superior pose estimation accuracy and robustness in highly dynamic indoor environments.

The main contributions of this work are summarized as follows:

1. A biologically-inspired visual SLAM framework is presented, which integrates instance segmentation and geometric feature fusion. The YOLOv8-seg model is employed to achieve precise contour-level detection of dynamic objects, providing semantic priors for robust dynamic region filtering.
2. An angle-based filtering module is developed based on LSD-extracted line features, and an adaptive-weight reprojection error function is introduced. By jointly optimizing point and line constraints, the accuracy and robustness of camera pose estimation are significantly improved in dynamic environments.
3. The proposed framework is implemented as a complete system, IPL-SLAM, and its effectiveness is validated on the TUM RGB-D dataset. The system demonstrates superior localization accuracy and high-quality semantic mapping compared to existing dynamic SLAM methods.

## 2. Related Work

### 2.1. Visual SLAM

Visual SLAM has gained the attention of researchers, especially in terms of its affordability and compactness, making it a promising option for various applications. In 2007, Davison A. J. proposed Mono-SLAM [16], which was the first monocular real-time SLAM system. This groundbreaking work not only expanded the range of robotic systems where SLAM can be applied successfully but also opened up new avenues for research and development in the field. Following this, Klein introduced PTAM [17] (Parallel Tracking and Mapping), which was designed to split the front-end of SLAM into two threads. One thread focused on estimating the camera posture while the other was responsible for mapping by restoring 3D information of feature points. Additionally, the keyframes strategy was implemented during the mapping and tracking processes, using keyframes to estimate camera pose, which led to significant improvements in both mapping and tracking accuracy. Forster introduced a fast Semi-direct Monocular Visual Odometry (SVO) [9] that incorporated both feature point and direct tracking optical flow methods. Subsequently, other frameworks such as Direct Sparse Odometry (DSO) [8] have been developed one after the other. Unlike traditional feature-based methods, DSO uses a direct approach, which means it directly minimizes the photometric error between the observed image intensities and the predicted intensities from the reconstructed scene. This allows for more accurate and dense reconstructions, especially in textured and dynamic environments. ORB-SLAM leverages the ORB (Oriented FAST and rotated BRIEF [18]) feature points [19] to establish correspondences between two consecutive frames, thereby enabling the calculation of camera pose. The algorithm employs bundle adjustment in its back-end to optimize the estimated pose and map points. Additionally, the loop closing module converts images to words using a bag of words approach, followed by global optimization using an optimization method. To improve efficiency, ORB-SLAM2 [5] divides the processing pipeline into separate threads for tracking, mapping, and loop closing. The tracking thread continuously estimates the camera pose and updates the map, while the mapping thread creates new map points and manages the 3D map. The loop closing thread detects previously visited locations and closes loops in the map to reduce drift and improve accuracy.

Despite the various methods currently available for SLAM systems, there are limitations when it comes to distinguishing between features in static and dynamic objects. This can result in inaccurate data association and errors in motion estimation, ultimately leading to a deterioration of the SLAM system’s performance. As a result, there is a need for further exploration and development of techniques that can better handle the challenges posed by dynamic environments.

To ensure that the novelty of IPL-SLAM is evaluated against the most recent developments, we have incorporated additional references to nascent works such as Transformer-based SLAM pipelines [20], lightweight dynamic object filtering methods [21], and YOLOv8-driven semantic SLAM systems [22]. These comparisons highlight that while recent approaches focus on either semantic segmentation or geometric constraints in isolation, IPL-SLAM’s integration of instance-level segmentation with adaptive point–line fusion achieves a better balance between real-time performance and dynamic feature suppression.

### 2.2. Dynamic VSLAM

In most SLAM systems, the environment is assumed to be static. This means that dynamic objects are not taken into account in the map or used in the tracking process. However, in real-world applications involving dynamic environments, this assumption is no longer valid. To address this issue, researchers have recently proposed several solutions. These solutions can be broadly classified into two main categories: geometric-based approaches and semantic-based approaches.

Geometric-based approaches use mathematical models to detect and track moving objects in the environment. These methods typically rely on visual odometry or motion estimation techniques that estimate the 3D motion of objects based on their 2D projections in the camera image. Fang et al. [23] employed optimum-estimation and uniform sampling techniques to detect dynamic objects, while Li et al. [24] utilized frame-to-keyframe registration and static point weight to minimize the adverse effects of moving objects. Sun et al. [25] proposed an enhanced RGB-D SLAM method that serves as a pre-processing stage to filter out data linked with mobile objects. Mu et al. [26] introduced an innovative feature selection algorithm in a dynamic environment, which was integrated into MSCKF based on trifocal tensor geometry. Xu et al. [12] devised a fresh visual odometry algorithm that eliminates moving objects by amalgamating spatial geometric information from the image and relying on the residual features to approximate the camera’s position. In addition, Wang et al. [13] suggested a novel RGBD SLAM approach with moving object detection for dynamic indoor environments, which is predicated on mathematical models and geometric constraints and can be assimilated into the SLAM process as a data filtering stage. Nevertheless, due to the paucity of geometric information, most geometric-based dynamic SLAM methods fail to attain optimal performance.

Semantic-based approaches, on the other hand, incorporate semantic information about the environment to detect and track dynamic objects. This involves using deep learning techniques to classify objects in the scene and predict their motion over time. By leveraging semantic information, these approaches can handle complex scenarios where objects may be partially occluded or have similar appearance. Elayaperumal et al. [27] proposed a semi-supervised video object segmentation framework named OSOSM, which leverages semantic knowledge from pre-trained models and specific object features from a single annotated frame to achieve efficient and accurate segmentation in dynamic and complex scenes. This method demonstrates superior performance compared to existing techniques, especially on benchmarks such as DAVIS and YouTube-VOS. Zhang et al. [28] utilized YOLO [29,30,31] to identify objects present in the environment and created a semantic map to filter dynamic feature points, resulting in improved system accuracy. Zhong et al. [21] presents a dynamic point-line SLAM method based on lightweight object detection, designed for real-time localization and mapping in dynamic environments. Xu et al. [32] propose a deep learning-based visual SLAM approach tailored for indoor dynamic scenes, leveraging semantic information to improve localization and mapping performance in dynamic environments. Zhu et al. [33] presents Ellipsoid-SLAM, an innovative approach that leverages an ellipsoid model to significantly enhance object detection and mapping capabilities in dynamic environments. By incorporating advanced geometric modeling and probabilistic reasoning, Ellipsoid-SLAM effectively addresses the challenges posed by moving objects and changing scenes, thereby improving the robustness and accuracy of SLAM systems in real-world applications.

Meanwhile, Bescos et al. [14] proposed a semantic system called DynaSLAM, based on ORBSLAM2, which uses Mask R-CNN [34] to segment every image frame and obtain a semantic mask. This approach integrates multi-view geometry and semantic mask to eliminate dynamic feature points and fill in background areas obscured by dynamic objects. Similarly, Liu et al. [35] employed various semantic segmentation techniques to detect dynamic objects and remove outliers. On the other hand, Yu et al. [15] introduced DS-SLAM, which mitigates the impact of dynamic objects through a combination of semantic segmentation and an optical flow method. While deep learning can be a powerful tool, it relies heavily on network quality. In some cases, networks with simple architectures may not be able to accurately identify dynamic objects, while more complex architectures can slow down system operation speed. It is important to keep these considerations in mind when selecting a suitable deep learning approach for a given application.

Despite the impressive performance of combining deep learning networks with SLAM systems to eliminate the impact of dynamic objects, there still exist some errors in the detection of dynamic object edges. These errors result in the loss of certain static features, preventing the complete elimination of surface feature points on dynamic objects. These inaccuracies not only decrease the accuracy of camera pose estimation but also lead to a decline in the quality of map construction. In this paper, we introduce IPL-SLAM to address the challenges posed by dynamic indoor scenes. To mitigate errors in the segmentation of dynamic object edges, we employ YOLOv8 for pixel-level instance segmentation. Furthermore, IPL-SLAM takes full advantage of line features in dynamic indoor scenes and introduces an error function with adaptive weights for different dynamic environments. As a result, our system improves the accuracy of camera pose estimation and enhances system robustness in dynamic indoor environments, contributing to better map construction.

## 3. System Introduction

**Figure 2:**
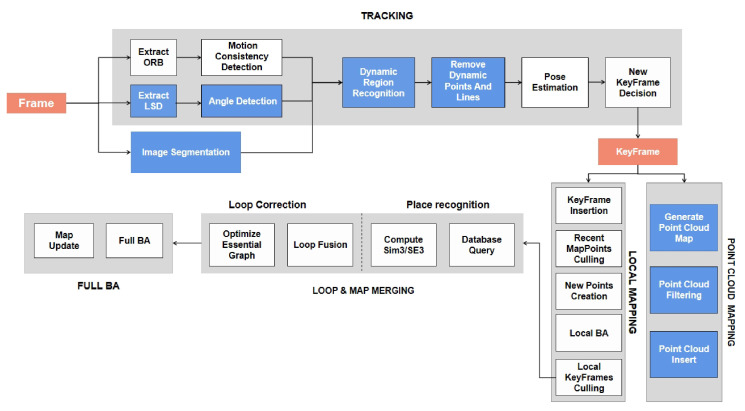
Component architecture of IPL-SLAM tracking and mapping backend. The pipeline is divided into three main stages: (1) Tracking: ORB and LSD feature extraction, motion and angle consistency detection, dynamic region recognition, and removal of dynamic points/lines before pose estimation and keyframe selection. (2) Local mapping: Keyframe insertion, point cloud generation, filtering, and bundle adjustment for local consistency. (3) Loop and map merging: Place recognition via database query, Sim3/SE3 computation, and essential graph optimization to maintain global map accuracy. Modules highlighted in blue indicate newly proposed components in IPL-SLAM.

To clarify the flow of the methodology, Figure 1 and Figure 2 provide a step-by-step overview of the IPL-SLAM pipeline. Figure 1 highlights the high-level data flow from RGB-D input to pose estimation and static map generation, while Figure 2 illustrates the modular structure of segmentation, feature extraction, dynamic feature filtering, and pose optimization in the tracking and mapping threads. The IPL-SLAM system proposed in this paper is a novel SLAM approach that leverages a YOLOv8 pixel-level instance segmentation network to obtain prior regions. YOLOv8 is selected over models like Mask R-CNN due to its significantly faster inference speed and anchor-free design, which aligns well with the real-time requirements of SLAM. Moreover, YOLOv8 provides accurate instance-level masks and strong generalization performance across different environments, making it a practical choice for dynamic SLAM scenarios. Additionally, line features are introduced, and a novel error function is designed for pose optimization, aiming to enhance the accuracy and robustness of the entire SLAM system. This section aims to provide a detailed explanation of the IPL-SLAM system, including its overall architecture and how it works.

### 3.1. Overview of IPL-SLAM

DS-SLAM, a classic dynamic SLAM system integrated with deep learning, employs ORB-SLAM2 as its framework. It combines semantic segmentation with motion consistency checking to remove dynamic objects and improve pose estimation. Unlike DS-SLAM, our IPL-SLAM leverages YOLOv8 for more accurate instance segmentation and introduces line features with an adaptive-weight error function.

The overview of this system is shown in Figure 2. Firstly, in the IPL-SLAM system, when processing each RGB image prior regions are extracted using the YOLOv8 instance segmentation network, while point features are obtained through the ORB extractor, and line features through the LSD extractor. Subsequently, by leveraging motion consistency checks for point features and angle checks for line features, combined with the concept of segmented regions, each point and line are classified as either dynamic or static. Secondly, based on the prior regions obtained from the YOLOv8 instance segmentation network, along with dynamic feature points and feature lines, dynamic regions can be identified by applying predefined thresholds. Subsequently, features within dynamic regions, including points and lines, are filtered out. Finally, the remaining static feature points and feature lines are used to calculate the dynamic–static weights for all points and lines. This information, combined with the reprojection errors of points and lines, is utilized to optimize the camera pose. The optimized pose, along with the depth image, is then employed to generate a static point cloud map.

### 3.2. YOLOv8 Instance Segmentation

Given the need for pixel-level dynamic object detection and semantic information mapping, this paper utilizes the YOLOv8 instance segmentation model pre-trained on the MS COCO dataset. The model is capable of segmenting 80 classes of commonly found objects in daily life [36].

YOLOv8 stands out in image segmentation due to its superior performance in handling occluded objects and producing smoother edges compared to other models. Its advanced segmentation capabilities enable more accurate detection of objects even when they are partially obscured, making it particularly effective in complex and dynamic environments. Additionally, YOLOv8 excels in edge processing, producing more natural and precise boundaries that enhance the quality of segmentation masks. These advantages make YOLOv8 an ideal choice for applications requiring high-precision segmentation, such as dynamic object detection in real-time systems. In the proposed IPL-SLAM system, YOLOv8 is employed to extract instance segmentation regions from input images, providing prior regions for dynamic object detection and semantic information. This integration not only enhances pose estimation accuracy and system robustness in dynamic environments but also allows for the creation of a static point cloud map by incorporating semantic information from segmented static objects.

**Figure 3:**
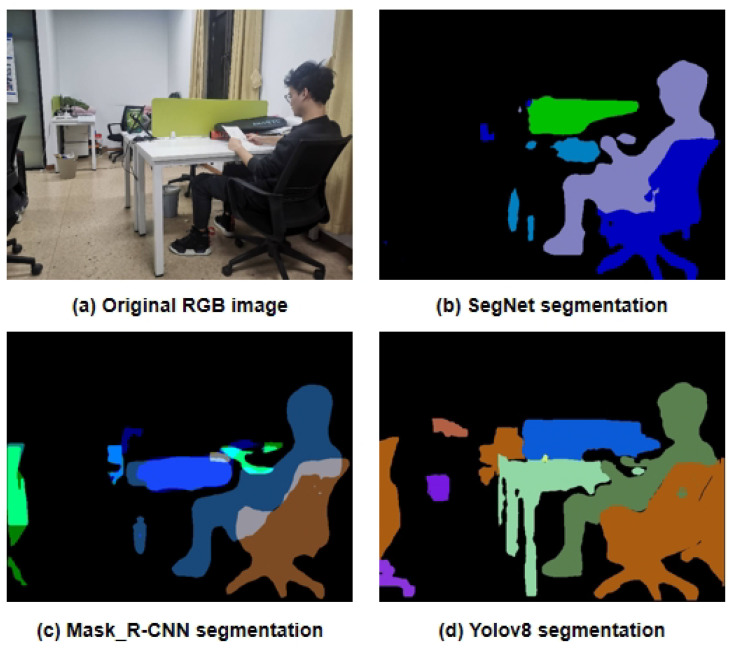
Comparison of different semantic segmentation networks for dynamic object detection.

In terms of segmentation effectiveness and detail processing, YOLOv8 outperforms in comparison to SegNet [37] and Mask R-CNN [34], with clearer and more complete mask edges obtained by YOLOv8, as illustrated in Figure 3. As illustrated in Figure 3, we compare the performance of SegNet, Mask R-CNN, and YOLOv8 for dynamic object segmentation. In the figure, (a) shows the original RGB input image, (b) presents the segmentation results of SegNet, which suffers from coarse boundaries and insufficient instance differentiation, (c) shows the output of Mask R-CNN, which provides precise masks but incurs higher computational cost, and (d) displays the result from YOLOv8, which achieves a good balance between segmentation accuracy and inference speed, making it ideal for real-time SLAM applications.

### 3.3. Dynamic Feature Filtering in Images Under Continuous Image Sequences

The segmentation masks obtained through the aforementioned experiments can only be identified as potential dynamic objects. To determine whether or not an object is a true dynamic object, it is necessary to assess its movement status within a certain time window across consecutive image frames. Therefore, this paper proposes a module based on a sliding window approach and contour similarity analysis for comparison, which is used to determine the movement status of objects across consecutive image frames.

**Figure 4:**
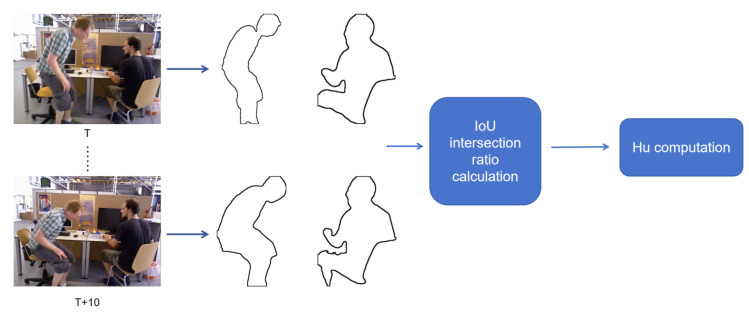
Workflow of contour similarity analysis used for dynamic object recognition. For each frame pair (T,T+10), object contours are extracted from semantic masks. The similarity between consecutive contours is computed using IoU (Intersection over Union) to measure overlap stability and Hu invariant moments to capture global shape characteristics. This combination allows robust detection of subtle dynamic motions while remaining efficient for real-time processing.

As shown in Figure 4, the contours of the mask are first extracted, and then a similarity analysis is performed on the contour images. Considering the real-time requirements of the SLAM system, this paper uses a combination of IoU (Intersection over Union) [38] and Hu invariant moments [39] to calculate the similarity of contours, and this module runs in a separate thread. IoU provides a simple and computationally efficient measure of overlapping regions, which is robust to small boundary fluctuations, while Hu invariant moments capture the global shape characteristics and remain stable under rotation, translation, and scaling. The combination of these metrics achieves a good balance between real-time performance and robustness compared to alternatives such as Chamfer distance or pixel-wise correlation metrics, making them well-suited for dynamic object filtering in real-time SLAM systems.For the identification of dynamic objects in consecutive image frames, a sliding window mechanism is adopted to store the continuous image sequence. In our implementation, the window interval is set to 20 frames, meaning that the system compares the current frame with a buffer of the previous 20 frames. This allows the algorithm to capture both short-term and slightly delayed object motions, smoothing out transient noise while maintaining real-time performance.

**Figure 5:**
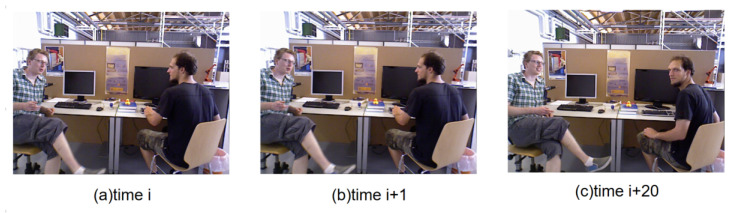
Illustration of temporal frame differences used for dynamic feature detection. Consecutive RGB-D frames at times *i*, i+1, and i+20 are shown to highlight subtle and large-scale motions. These temporal variations are used in IPL-SLAM to distinguish static background features from moving objects in low- and high-dynamic scenarios.

Figure 5 illustrates the differences between adjacent image frames and interval image frames. It can be observed from the figure that there is a very small difference between the adjacent image frames at time *i* and time *i* + 1. After an interval of 20 frames, it can be clearly seen that the person on the right has turned his head to the left. Therefore, using the data of interval image frames can effectively determine whether or not the object is in motion. For the setting of the sliding window length, if it is set too small it cannot effectively distinguish the motion state of the object; if it is set too large there will be significant differences between the objects and the background in the two frames, making it impossible to effectively perform pose estimation. In this paper, the window interval is set to 20, and combined with the above contour similarity analysis it can effectively identify potential dynamic objects, thus filtering out dynamic features. In this study, the window interval is set to 20 frames, which defines the temporal range over which contour similarity is evaluated. A larger interval enables detecting slow or subtle motions across a longer time span, while still maintaining computational feasibility for real-time operation.

**Figure 6:**
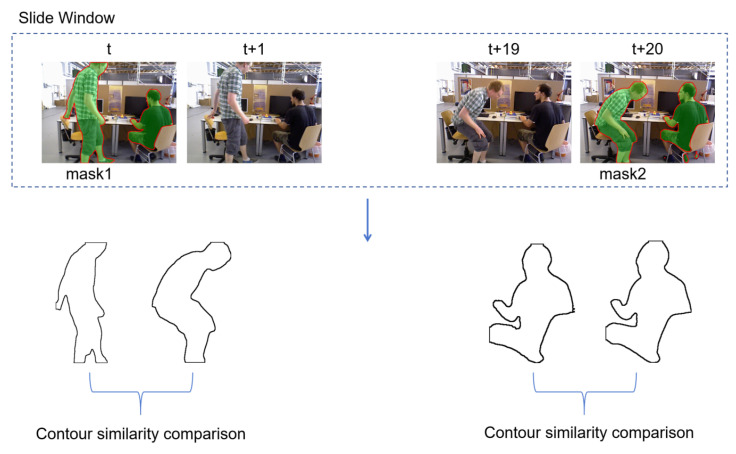
Dynamic object discrimination using a sliding window approach. Consecutive frames (*t* to t+20) are segmented to generate instance masks (mask1 and mask2), and the contours of detected objects are extracted for similarity analysis. By comparing contour consistency across short and long temporal gaps, IPL-SLAM distinguishes truly static objects from low-motion dynamic elements such as slight body movements or hand gestures.

In summary, through the semantic segmentation network, the mask of the object can be obtained. Subsequently, by conducting a contour similarity analysis, the motion status of the object can be determined. Thus, feature points belonging to dynamic objects can be eliminated, and the remaining static feature points can be used for pose estimation. As shown in Figure 6, the process of identifying moving objects is presented.

In the figure, the image at the head of the sliding window corresponds to time *t*, representing the object mask obtained via the semantic segmentation network. Similarly, the image at the tail of the window corresponds to time *t* + 20, depicting the mask image acquired at that moment. The entire sliding window contains 20 consecutive frames of images. Based on the contours of the masks, a similarity analysis can be performed using the aforementioned algorithm. In Figure 6, the person standing on the left exhibits significant changes, resulting in a lower calculated similarity, whereas the person sitting on the right shows no obvious movement, only slightly turning their head, which leads to a higher calculated contour similarity. Ultimately, it can be determined that the person on the left is a moving object, and all feature points on them across the sliding window images need to be filtered out. In contrast, the person on the right is deemed relatively stationary, and their feature information can be retained for pose estimation.

Therefore, as long as potential dynamic objects are identified within the sliding window, all the masks corresponding to this dynamic object in all frames within the sliding window will be marked as dynamic. Moreover, the potential dynamic objects identified in the nearest key frames are marked, in preparation for the subsequent algorithms. In addition to the qualitative results shown in Figure 3, Figure 4, Figure 5 and Figure 6, quantitative evaluations on the TUM RGB-D dataset are presented in Table 1 and Table 2, where IPL-SLAM achieves consistent improvements in ATE/RMSE compared to baseline methods.

In future experiments, we will introduce a dynamic object record table in the form of a hash map to track the categories of dynamic objects detected in the current frame through sliding window analysis and contour similarity analysis. Before conducting dynamic region analysis in subsequent frames, the system first checks whether or not the object categories detected by the image segmentation algorithm are already present in the hash map. If they are, the category is directly classified as dynamic, bypassing the sliding window and contour similarity analysis steps. If not, dynamic object analysis continues, and the hash map is updated in real time.

This approach not only reallocates computational resources more efficiently to other tasks, thereby reducing execution time and improving system performance, but also reduces model complexity over long sequences, thereby minimizing the risk of classification errors.

### 3.4. Remove Dynamic Point and Line Features

As mentioned in the previous section, the sliding window approach and contour similarity analysis can only roughly identify dynamic objects. Contour features are significantly affected by factors such as lighting and changes in the background environment. To address this issue, this study conducts point and line feature extraction on the dynamic objects identified in the previous section, thereby achieving more refined and accurate removal of dynamic objects and reducing the impact of dynamic features on accuracy. In addition, considering that line feature extraction and matching are relatively time-consuming, all line feature operations in this study are carried out in a separate thread and are performed only on key frames. For ordinary frames, only point feature extraction is conducted.

Initially, line feature extraction is performed within key frames (i.e., image frames with overlapping fields of view). Subsequently, by leveraging the motion consistency of feature points and the epipolar constraint algorithm, the distance from feature points to epipolar lines is precisely calculated. Points exceeding a predefined threshold are identified as potential dynamic feature points. Concurrently, the IPL-SLAM system incorporates an angle detection module to identify dynamic feature lines. This module is based on the optical flow method, which assumes that all feature points in the image share identical motion in a static environment—an assumption that also applies to line features. A reasonable assumption is made: for successfully extracted line features, under conditions where there is minimal visual change and objects do not undergo drastic motion, the 3D positions of their endpoints remain constant throughout the tracking process. With this assumption in place, line features can be processed similarly to point features and combined with the optical flow method.

**Figure 7:**
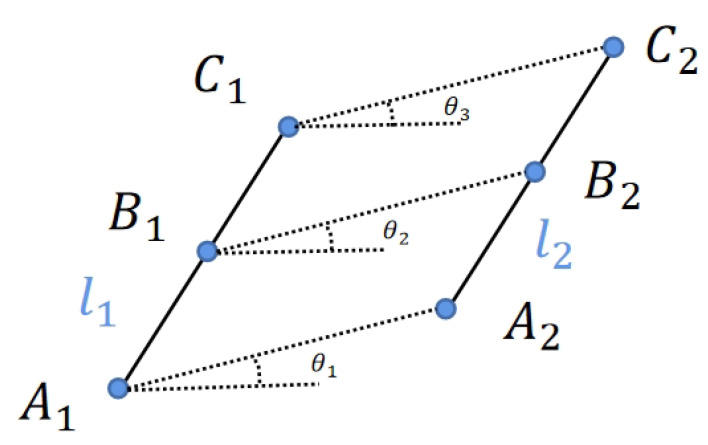
The illustration of optical flow selection.

As depicted in Figure 7, line segments $l_{1}$ and $l_{2}$ are feature lines that have been matched by the LBD algorithm in consecutive frames of images. Points $A_{1}$, $B_{1}$, and $C_{1}$ represent the starting point, midpoint, and endpoint of $l_{1}$, respectively, while points $A_{2}$, $B_{2}$, and $C_{2}$ correspond to those of $l_{2}$. According to our assumption, if the line feature is dynamic, then, within the same image motion, the angles between the corresponding three trajectories of these points and the X-axis, denoted as $\theta_{1}$, $\theta_{2}$, and $\theta_{3}$, are expected to be identical. The average of the differences in these three angles is calculated. If this value surpasses a predefined threshold, the line feature is deemed to be dynamic.

Thus far, we have successfully identified dynamic feature points and feature lines within key frames. By leveraging the potential dynamic objects preliminarily identified through contour analysis and processing them in accordance with Algorithm 1, we can more precisely determine the final dynamic regions in the continuous image sequence.

Firstly, initialize two empty sets, $S_{1}$ and $S_{2}$, to store dynamic regions. Next, traverse all prior regions *M*, tallying the count of dynamic feature points and feature lines in each region. Subsequently, based on the count of dynamic feature points in a region, determine if it qualifies as a potential dynamic region, adding it to set $S_{1}$. Similarly, based on the count of dynamic feature lines in a region, assess its potential as a dynamic region and include it in set $S_{2}$. Finally, filter out regions that appear in both sets $S_{1}$ and $S_{2}$, identifying them as the ultimate dynamic regions. Then, utilizing the mask of dynamic regions, filter out dynamic feature points and feature lines.
**Algorithm 1** Dynamic region recognition**Require:** Set of prior regions *M* in current frame; Set of matched points $P_{1}$, $P_{2}$; Set of matched lines $L_{1}$, $L_{2}$**Ensure:** Dynamic area mask $M_{d}$1:Initialize $S_{1}\leftarrow \emptyset$2:Initialize $S_{2}\leftarrow \emptyset$3:**for** each region *m* in *M* **do**4:    $n_{p}\leftarrow 0$5:    **for** each $\left(p_{1},p_{2}\right)$ in $P_{1}$, $P_{2}$ **do**6:        **if** $p_{1}$ not in *m* or $p_{2}$ not in *m* **then**7:           **continue**8:        **end if**9:        $F_{M}\leftarrow \mathrm{ComputeFundamentalMatrix}\left(P_{1},P_{2}\right)$10:        $l_{1}\leftarrow \mathrm{FindEpipolarLine}\left(p_{1},F_{M}\right)$11:        $D\leftarrow \mathrm{CalcDistanceFromEpipolarLine}(p_{2},l_{1})$12:        **if** D>threshold **then**13:           $n_{p}\leftarrow n_{p}+1$14:        **end if**15:    **end for**16:    **if** $n_{p}>\mathrm{threshold}$ **then**17:        $S_{1}\leftarrow S_{1}\cup \left\{m\right\}$18:    **end if**19:    $n_{l}\leftarrow 0$20:    **for** each $\left(l_{1},l_{2}\right)$ in $L_{1}$, $L_{2}$ **do**21:        **if** $l_{1}$ not in *m* or $l_{2}$ not in *m* **then**22:           **continue**23:        **end if**24:        $\theta_{\mathrm{avg}}\leftarrow \mathrm{CalcAngleFromLine}\left(l_{1},l_{2}\right)$25:        **if** $\theta_{\mathrm{avg}}>\mathrm{threshold}$ **then**26:           $n_{l}\leftarrow n_{l}+1$27:        **end if**28:    **end for**29:    **if** $n_{l}>\mathrm{threshold}$ **then**30:        $S_{2}\leftarrow S_{2}\cup \left\{m\right\}$31:    **end if**32:**end for**33:**for** each region *m* in *M* **do**34:    **if** $m\in S_{1}$ and $m\in S_{2}$ **then**35:        $M_{d}\leftarrow M_{d}\cup \left\{m\right\}$36:    **end if**37:**end for**38:**return**$M_{d}$

**Figure 8:**
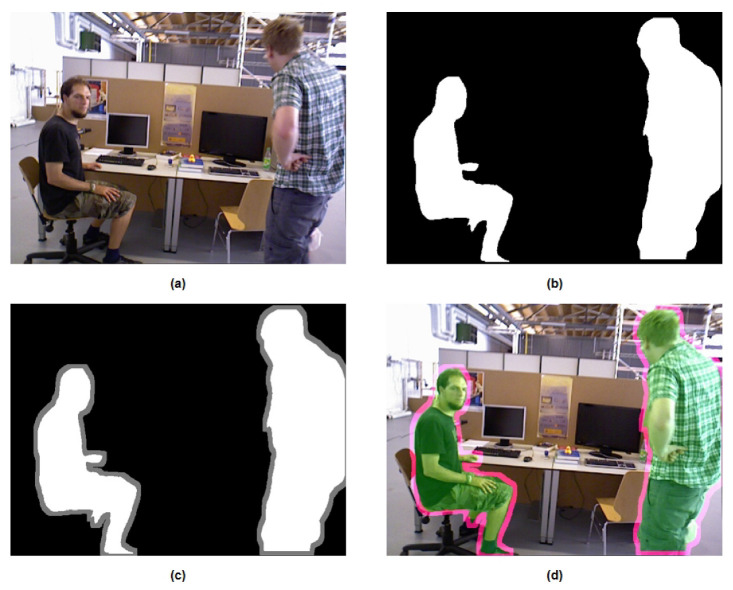
Expansion of the mask.

**Figure 9:**
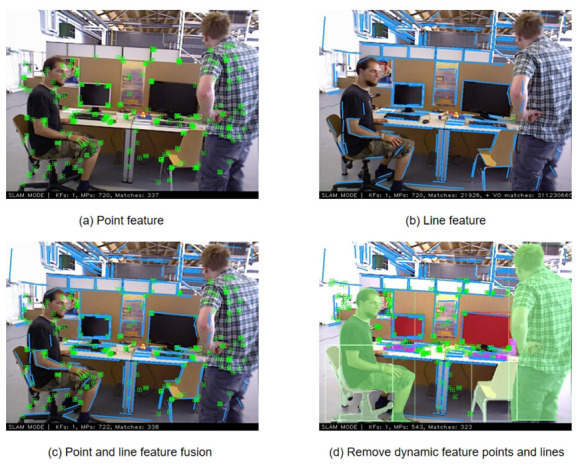
Removal of dynamic features.

However, when extracted key points and feature lines happen to be right on the edge of the mask, it becomes challenging to filter them out. In order to address this issue, IPL-SLAM employs an extension process for the mask. It initially detects pixels with RGB values of (255,255,255) in a frame from the semantically segmented image. Subsequently, it sets the pixel values within a circular region centered around that pixel, with a radius of ten pixels, to (255,255,255). This results in an expanded mask, as illustrated in Figure 8, where (a) represents the original image, (b) depicts the mask obtained through YOLOv8, (c) showcases the expanded mask, and (d) displays the overlay of the mask on the original image. It is evident that the edges of dynamic objects have been extended, allowing for the removal of key points and feature lines that originally resided on the edges. As depicted in Figure 9, Figure 9a showcases feature points extracted using ORB, Figure 9b depicts line features extracted through LSD, Figure 9c represents the fused feature map of points and lines, while Figure 9d shows the remaining static feature points and lines after filtering out the dynamic ones.

### 3.5. Optimization Model Based on Static Point and Line Feature

After removing dynamic features, the remaining points and lines are considered static. The camera pose can be iteratively computed by constructing a geometric optimization model. In this study, we project the features from the previous frame, including points and lines, into three-dimensional space. Subsequently, based on the initial pose from a constant velocity motion model, we reproject the 3D points and lines onto the current frame. Throughout this process, the reprojection errors of points and lines can be treated as a function of the pose. Therefore, the optimal camera pose can be determined through iterative optimization.

In the previous frame, the three-dimensional coordinates of a certain feature point *p* are denoted as $P_{w}$. With the initial camera pose set as *T* based on a constant velocity motion model, and *K* representing the camera intrinsic parameters, the pixel coordinates $P_{uv}$ are obtained by reprojecting $P_{w}$ onto the current frame using the following formula:

(1) $$\begin{array}{c} P_{uv}=f\left(P_{w},K,T\right) \end{array}$$

In the current frame, the pixel coordinates $P_{uv}^{\prime }$ for the matched point $p^{\prime }$ corresponding to a point *p* are determined using a feature matching algorithm. The reprojection error of the feature point is then computed by measuring the distance between the matched point and the reprojected point, as defined by the following formula:

(2) $$\begin{array}{c} E_{j}=P_{uv}^{\prime }-P_{uv} \end{array}$$

For each detected point feature $P_{uv}^{\prime }$ in the image plane, its corresponding 3D world coordinate $P_{w}$ is obtained by back-projecting the pixel into the camera frame using the depth value D(u,v) and the intrinsic calibration matrix *K*:

$$P_{c}=D\left(u,v\right)K^{-1}\left[\begin{array}{c} u\\ v\\ 1 \end{array}\right],$$

where $P_{c}$ is the 3D point in the camera coordinate system. The transformation to the world frame is then achieved via the estimated camera pose (R,t):

$$P_{w}=RP_{c}+t.$$

The reprojection error $E_{j}$ is computed by projecting $P_{w}$ back into the image and minimizing the difference between the observed feature $P_{uv}^{\prime }$ and the reprojected location.

**Figure 10:**
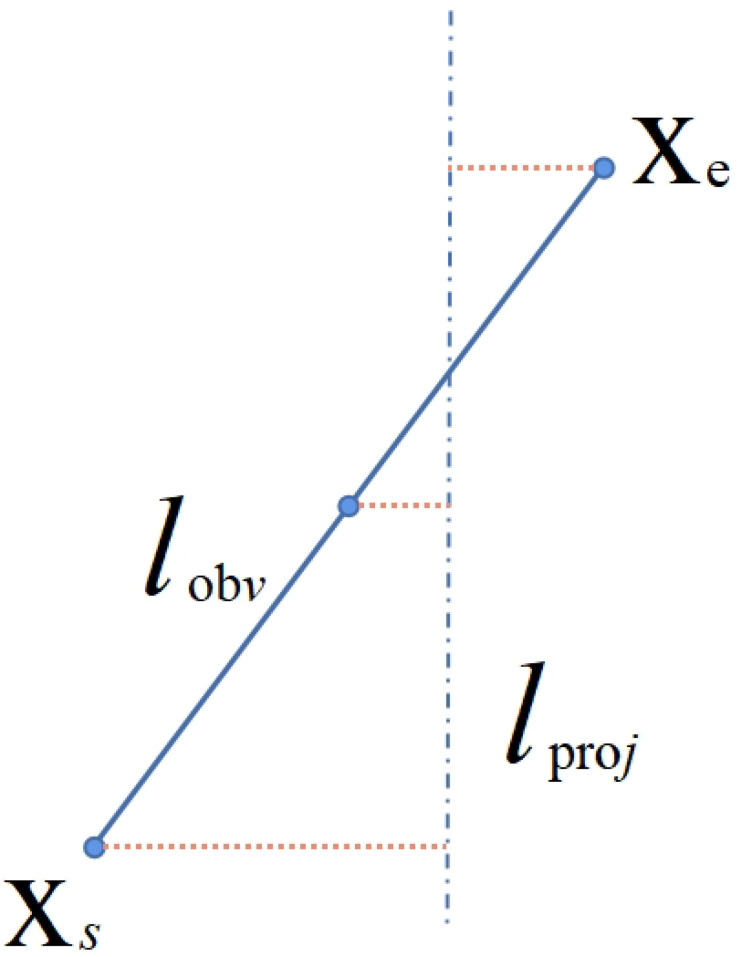
The reprojection of the line.

In comparison to feature points, the reprojection error for lines is somewhat more intricate. After completing the matching of line features, based on the corresponding depth map from the previous frame, the respective 3D coordinates of the line features in the previous frame are computed. Subsequently, as illustrated in Figure 10, the two endpoints of the 3D line features are projected onto the current frame. The distance of each endpoint from the projected line is then calculated, constructing a reprojection error model. Here, $X_{e}$ represents the endpoint of the observed line $l_{obv}$, $X_{s}$ is the starting point, and $l_{proj}$ is the matched line obtained through line feature matching. Ultimately, the reprojection error for the current frame’s line features can be expressed by the following formula:

(3) $$\begin{array}{c} E_{l}=\left[\begin{array}{c} d(X_{s},l^{\prime })\\ d(X_{e},l^{\prime }) \end{array}\right]=\left[\begin{array}{c} \frac{X_{s}^{T}l^{\prime }}{\left|\right|l^{\prime }\left|\right|}\\ \frac{X_{e}^{T}l^{\prime }}{\left|\right|l^{\prime }\left|\right|} \end{array}\right] \end{array}$$

where $l^{\prime }$ denotes the space line *l* projected on the 2D plane, $X_{e}$ and $X_{s}$ represent the two endpoints on the matching line, $X_{e}^{\prime }$ and $X_{s}^{\prime }$ denote their projection points on the line $l^{\prime }$, and $\left|\right|l^{\prime }\left|\right|$ represents the L2 norm of the vector $l^{\prime }$.

Combining the two aforementioned reprojection errors allows for the initial construction of an optimization problem, iterating to compute the optimal camera pose. However, this optimization problem relies heavily on the quality of extracted static feature points and lines. Incorrectly treating certain dynamic features as static points or lines in constructing the entire geometric model can result in substantial errors, significantly impacting pose estimation and reducing the overall localization accuracy of the system.

While we have filtered out a majority of feature points and lines from dynamic regions based on earlier image segmentation and geometric constraints, there are scenarios where certain dynamic features persist within locally moving or stationary regions. Consider a situation where a person seated in a chair turns their head or waves their hand; most of the person’s features remain static, with only a few dynamic features present around the head and hand. Failing to appropriately constrain optimization in such scenarios would inevitably introduce errors from these dynamic features into the optimization model.

**Figure 11:**
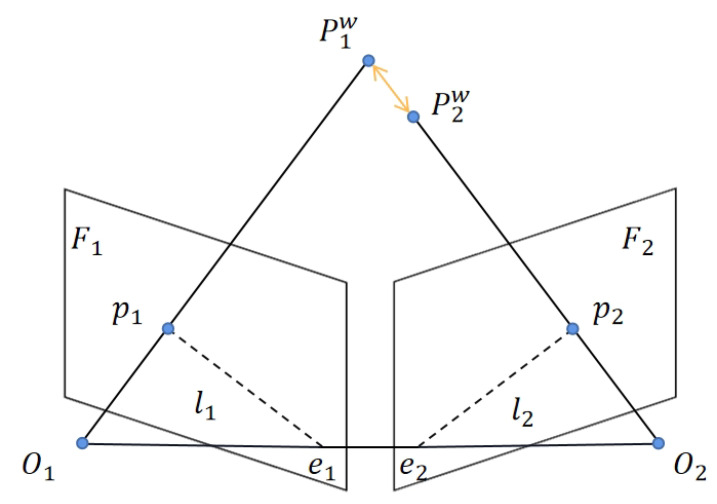
Epipolar constraint and dynamic environment. $\theta_{1}$, $\theta_{2}$, and $\theta_{3}$ represent the angular deviations between corresponding point-line features and the epipolar line, used to evaluate geometric consistency.

To address this issue, we have enhanced the original reprojection error function and introduced adaptive weighting coefficients for each feature, outlined in the following formula:

(4) $$\begin{array}{c} e=\sum_{j}\left(\alpha_{p}e_{j}^{T}\omega_{j}^{-1}e_{j}\right)+\sum_{l}\left(\alpha_{l}e_{l}^{T}\omega_{l}^{-1}e_{l}\right) \end{array}$$

where $e_{j}$ and $e_{l}$ represent the reprojection error for points and lines, respectively, while *e* denotes the error function that fuses point and line features. The weight coefficient $\alpha_{p}$ for the feature point is calculated in conjunction with a geometric model. Initially, we traverse the remaining static feature points in the image, computing the distance *d* between matched feature points in two frames based on epipolar constraints and motion consistency checks. As shown in Figure 11, $p_{1}$ and $p_{2}$ are pixel points on the image plane, whereas $P_{1}^{w}$ and $P_{2}^{w}$ are the 3D points obtained by projecting the pixel points according to the camera pose and intrinsic matrix. The disparity between them represents the sought distance *d*. Subsequently, sorting by distance allows us to determine the maximum distance $dist_{max}$ and minimum distance $dist_{min}$. Finally, the weight coefficient for the feature point is obtained using the following formula, where the coefficient range is from 0 to 1:

(5) $$\begin{array}{c} \alpha_{p}=\frac{dist_{max}-d}{dist_{max}-dist_{min}} \end{array}$$

Compared to traditional uniform weighting schemes, the adaptive-weighting mechanism explicitly models the uncertainty of individual features based on geometric consistency. By assigning lower weights to features with larger epipolar or angular deviations, the optimization problem becomes more robust to outliers introduced by residual dynamic elements. This approach is particularly effective in partially dynamic environments where static and dynamic regions coexist (e.g., seated individuals with minor limb motion). In such cases, uniformly weighting all features often propagates errors from local motion into global pose estimation, while the adaptive strategy suppresses these effects by emphasizing geometrically consistent static constraints. The method also provides a smooth transition between fully static and highly dynamic scenarios without requiring manual threshold tuning.

To ensure consistency, $\alpha_{p}$ is normalized to the range [0,1], and values outside this interval are clipped. An empirical threshold $\tau_{p}=0.05$ is applied: points with $\alpha_{p}<\tau_{p}$ are discarded from the optimization to suppress residual dynamic features. If the distance between matched feature point pairs is greater, it indicates a higher probability that the feature point is dynamic. Consequently, in subsequent optimization steps, its reprojection error is assigned a smaller weight coefficient.

Similarly, $\alpha_{l}$ represents the weight coefficient for feature lines, and its calculation method is akin to that of feature points. Initially, traverse the remaining static feature lines in the image. Then, based on previously computed angles $\theta_{1}$, $\theta_{2}$, $\theta_{3}$ from angle detection in Figure 7, we can obtain the average of the angle differences according to the following formula:

(6) $$\begin{array}{c} \theta_{average}=\frac{\left|\right|\theta_{1}-\theta_{2}\left|\right|+\left|\right|\theta_{1}-\theta_{3}\left|\right|+\left|\right|\theta_{2}-\theta_{3}\left|\right|}{3} \end{array}$$

Finally, the weight coefficient can be obtained according to the following formula:

(7) $$\begin{array}{c} \alpha_{l}=\cos\left(\theta_{average}\right) \end{array}$$

The coefficient $\alpha_{l}$ is inherently bounded within [0,1]. A small threshold $\tau_{l}=0.1$ is used to reject line features with very low stability before pose optimization. According to the assumption of optical flow, static feature points exhibit the same motion in two images. Therefore, the angles formed by the feature lines they constitute should be close to 0. As the average angle increases, the probability that the line feature is dynamic becomes higher. Consequently, the weight assigned to the average angle after cosine computation becomes smaller. The cosine operation also ensures that the weight coefficient remains within the range of 0 to 1.

**Figure 12:**
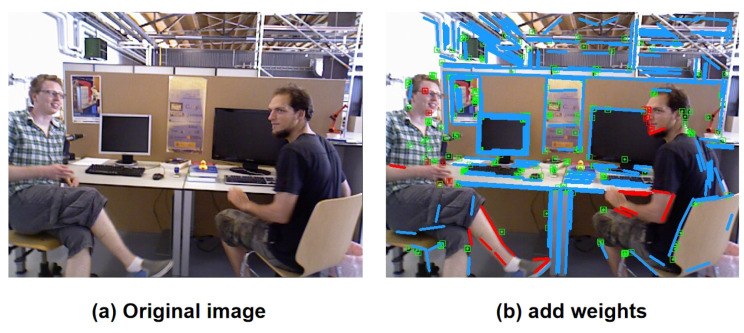
Feature points and feature lines after adding adaptive weights.

For ease of observing the effects after introducing adaptive weights, we visualized point and line features with different weights, as depicted in Figure 12, where Figure 12a represents the original image, and Figure 12b illustrates the point and line features extracted with the addition of adaptive weights. The red-colored features indicate points and lines with smaller weight coefficients during the pose optimization process, implying a higher probability of being associated with dynamic elements.

In the case of Figure 12, for the two individuals seated on chairs, based on the dynamic region identification algorithm discussed in the previous section, within the sliding window of the current frame, since the two individuals on the chairs exhibit no significant movement during the current time period, only slight movements in their heads and hands, they are deemed as static objects through contour similarity analysis, and no mask expansion is performed on them. In our implementation, a dynamic object is identified when the average contour similarity within the sliding window drops below 0.85 or when the proportion of feature points exceeding the epipolar distance threshold reaches 20%. Slight local movements, such as minor head rotations or hand gestures that keep the similarity above this threshold, are thus treated as static to preserve stable features for pose estimation. Subsequently, after point and line feature extraction of the entire image, according to the adaptive weighting algorithm proposed in this section, most features are classified as static. However, for the person on the left, local dynamic features are displayed in the head, hands, and legs; similarly, for the person on the right the head and hands also show local dynamic features. Therefore, in the IPL-SLAM system, features in these regions are assigned smaller weights, thereby reducing their impact in the subsequent pose optimization process. This strategic adjustment enables the system to more effectively utilize fully static features, thus achieving more accurate pose estimation. Moreover, the introduction of the adaptive weighting algorithm also serves as a final filter for any erroneous filtering in the dynamic region filtering algorithm mentioned earlier.

It is worth noting that the weight coefficient $\alpha_{l}$ is not obtained through any pre-training or offline learning process. Instead, it is computed adaptively at a runtime based on the geometric consistency of line features, using the angular deviation between corresponding segments and the epipolar line. This makes the approach independent of dataset-specific training and suitable for online SLAM scenarios. Since $\alpha_{l}$ is derived from instantaneous feature geometry rather than temporal patterns, it is not directly used to infer future motion but rather to robustly suppress dynamic outliers in the current frame.

## 4. Experiments

In this paper, we validate the performance of the developed IPL-SLAM using the TUM RGB-D public dataset. Firstly, IPL-SLAM is compared to the original DS-SLAM to verify the performance improvement of the developed approach. Secondly, we conduct an analysis of IPL-SLAM in comparison to other dynamic SLAM methods. Subsequently, we perform ablation experiments and time evaluations on IPL-SLAM to validate the effectiveness of the developed system. Finally, experiments are conducted in real-world scenarios to assess the practical application performance of IPL-SLAM.The open-source assessment tool by the TUM computer vision group is utilized to evaluate the performance of our method. The metric indices of Absolute Trajectory Error (ATE) and Relative Pose Error (RPE) are employed to assess the effectiveness of our SLAM approach. The deviation between the actual and estimated trajectories can be expressed through the Root-Mean-Square Error (RMSE) of ATE, while the translational and rotational drift [40] is quantified by RPE. All experiments are executed on a laptop computer equipped with an Intel Core i7-8750H (2.20 GHz) CPU, 8 GB RAM, NVidia GTX 1050Ti GPU, and Ubuntu 22.04 system.

To assess the robustness of our results, each sequence was evaluated over multiple runs (n = 5), and the reported RMSE and ATE values represent the mean ± standard deviation. The TUM evaluation tool also provides translational and rotational drift statistics, which indirectly reflect the confidence interval of trajectory estimation. While the current study focuses on benchmarking against state-of-the-art methods, future work will include formal statistical significance tests (e.g., paired *t*-tests or Wilcoxon signed-rank tests) to further validate the observed improvements.

### 4.1. Experiments in TUM RGB-D Dataset

The TUM RGB-D dataset is a comprehensive collection of RGB-D data and ground-truth information designed to serve as a benchmark for evaluating visual odometry and VSLAM systems. This dataset includes color and depth images captured by a Microsoft Kinect sensor, accompanied by the corresponding groundtruth trajectory of the sensor. By providing such a rich set of data, the TUM RGB-D dataset allows researchers to rigorously test the performance of their algorithms and compare them against state-of-the-art methods. In the TUM dataset, the ground-truth trajectory was acquired using a high-accuracy motion-capture system consisting of eight high-speed tracking cameras recording at 100 Hz. In our experiments, the dataset belonging to the “fr3” series is primarily utilized. Specifically, we focus on the sequence labeled as “fr3/walking” (abbreviated as fr3/w/), which captures footage of two individuals walking through an office environment. Notably, these sequences represent high-dynamic environments. The fr3/w/ sequences included four camera types. The “xyz” component contains the trajectory of the objects in a 3D coordinate system, providing information on their position and orientation at each time step. The object has been manually moved along three axes (x, y, and z) without changing its orientation. Additionally, the camera motion during this movement is relatively minor. In “rpy”, “Roll, Pitch, and Yaw” (RPY) are the three angles that define the rotational position of the robot relative to its starting point. Roll describes the rotation around the x-axis, pitch describes the rotation around the y-axis, and yaw describes the rotation around the z-axis. In “half”, the camera has been moved on a small half sphere of approximately 1 m in diameter. This sequence is intended to evaluate the robustness of VSLAM and odometry algorithms to quickly move dynamic objects in large parts of the visible scene. In “static”, the sensor has remained in place to capture the images and depth information of a static scene. This means that the camera does not move during the recording, which makes it easier to estimate the camera’s position in 3D space. We mainly use fr3/w/xyz, fr3/w/rpy, fr3/w/half, and fr3/w/static to evaluate our method.

To further validate the performance of our developed system in dynamic indoor scenarios, we conducted quantitative analysis experiments using the frb3 sequence dataset. Evaluation metrics [40] included Root Mean Square Error (RMSE), Mean Error (Mean), Median Error (Median), and the Standard Deviation of the absolute trajectory (STD). Among these, RMSE and STD better reflect the accuracy and the robustness of the system. Quantitative comparison results are presented in the tables below, with values highlighted in bold indicating the optimal outcomes for camera pose estimation. Quantitative comparison results are presented in the tables below (Table 1, Table 2 and Table 3), with values highlighted in bold indicating the optimal outcomes for camera pose estimation.

Table 1 illustrates that our developed IPL-SLAM outperforms DS-SLAM in dynamic dataset sequences. Significant performance improvements are observed particularly in highly dynamic datasets such as fr3/w/xyz and fr3/w/rpy. The limitations in the semantic segmentation performance of DS-SLAM resulted in ineffective elimination of some dynamic points, thereby compromising the accuracy of camera pose estimation. To address this, our research leverages YOLOv8 instance segmentation, which offers enhanced segmentation capabilities, effectively resolving the segmentation shortcomings of DS-SLAM. Concurrently, in the fr3/w/xyz and fr3/w/rpy datasets, dynamic objects exhibit relatively large movements. Through the sliding window and contour similarity analysis algorithms mentioned earlier, dynamic objects can be effectively filtered out. Moreover, with the introduced loss function incorporating adaptive weights, some dynamic features can also be eliminated in environments with local motion features. As a result, our algorithm demonstrates its advantages in these two datasets. In contrast, in the fr3/w/static dataset, human movements are not significant, and the differences in the contours of the human masks between adjacent frames are minimal. Therefore, using the algorithm in Section 3.3, they are treated as static objects. Subsequently, adaptive weighting is employed to distinguish between dynamic and static features, making the advantage of our algorithm less pronounced in this dataset. Overall, the research results indicate that the IPL-SLAM system developed in this study performs exceptionally well in trajectory estimation and exhibits greater robustness in highly dynamic scenes. However, its advantage is not evident in low-dynamic sequences (such as the fr3/w/static sequence). This can be attributed to the fact that in low-dynamic scenes, the movement of dynamic objects is minimal, and the impact of removing dynamic features on the accuracy of camera pose estimation is relatively small.

The developed IPL-SLAM has been compared with representative dynamic SLAM systems, including DynaSLAM, CFP-SLAM [41], YOLO-SLAM [42], PLD-SLAM [43], DPL-SLAM [21], and ORB-SLAM2. Quantitative experimental results are presented in Table 2, with dashes indicating that corresponding data were not provided in the original literature. Among the five experimental data sequences, the camera pose estimation accuracy using our developed system has shown improvement compared to the ORB-SLAM2 system. In comparison with DynaSLAM, CFP-SLAM, YOLO-SLAM, Dynamic point-line SLAM, and PLD-SLAM, our developed system performs better on the fr3/w/xyz and fr3/s/rpy datasets, while exhibiting slightly suboptimal results on other datasets.

To further substantiate the performance of IPL-SLAM, we have included detailed quantitative comparisons with state-of-the-art dynamic SLAM systems using RMSE and ATE metrics across five TUM RGB-D sequences. The results in Table 2 and Table 3 demonstrate consistent improvements in camera pose estimation accuracy, particularly in high-dynamic scenarios, validating the effectiveness of the proposed approach. To better contextualize these results, we further discuss the limitations of competing methods and the runtime trade-offs of our system.

Methods such as DynaSLAM and YOLO-SLAM have demonstrated strong performance in dynamic environments but also exhibit notable limitations. DynaSLAM relies on Mask R-CNN for segmentation, producing accurate masks but with substantial computational overhead that challenges real-time operation. YOLO-SLAM achieves faster inference via lightweight detection but its bounding-box segmentation lacks contour precision, often leaving residual dynamic features. Similarly, CFP-SLAM focuses on feature selection without semantic awareness, limiting its ability to handle large-scale moving objects. In contrast, IPL-SLAM combines instance-level segmentation with geometric point–line fusion, balancing segmentation accuracy and runtime efficiency. By incorporating adaptive-weight optimization and fine-grained contour-based filtering, the proposed system addresses both the computational cost and boundary-precision issues observed in competing approaches, achieving a better trade-off between accuracy and real-time feasibility.

The relatively higher runtime of IPL-SLAM compared to existing methods is primarily due to the inclusion of YOLOv8-based instance segmentation and LSD line feature extraction, which provide richer semantic and geometric constraints at the cost of additional computation. This trade-off leads to improved pose estimation accuracy and dynamic feature suppression in challenging scenes, as reflected in the quantitative results. It is also worth noting that the current implementation uses an unoptimized PyTorch 2.8.0 pipeline. With hardware acceleration (e.g., TensorRT inference), model pruning, and lightweight segmentation backbones, the runtime can be significantly reduced for real-time deployment.

To further validate the performance of camera pose estimation utilizing instance segmentation and point-line features with adaptive weights, this study conducted ablation experiments on the TUM RGB-D dataset, employing the RMSE metric for absolute trajectory errors. According to the experimental results presented in Table 3, the proposed camera pose estimation system exhibits improved performance on the fr3/w/xyz, fr3/w/static, and fr3/w/rpy sequences when the LSD line feature algorithm and adaptive weights are incorporated, resulting in reduced ATE values. These findings indicate that the accuracy of camera pose estimation is enhanced by introducing instance segmentation and point-line features with adaptive weights.

**Figure 13:**
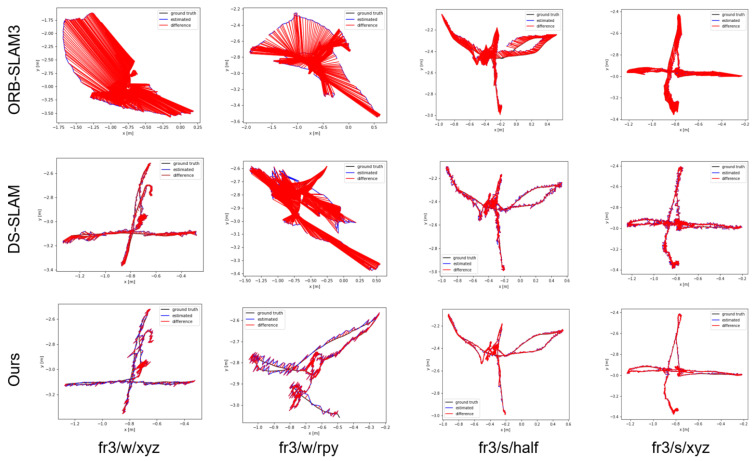
Comparison of the estimated trajectories.

Furthermore, as illustrated in Figure 13, the algorithm presented in this paper exhibits clear advantages in the four sequences in the figure. In the fr3/w/xyz and fr3/w/rpy datasets with high dynamics, the algorithm presented in this paper uses dynamic region analysis to filter out dynamically moving objects, such as people and chairs being dragged, thereby significantly improving the system’s localization accuracy. In contrast, in the fr3/s/half and fr3/s/xyz datasets with low dynamics, the algorithm employs adaptive-weight pose optimization to filter out dynamic features on partially dynamic objects. Compared to ORB-SLAM2 and DS-SLAM, the proposed algorithm also demonstrates certain advantages in localization accuracy.

### 4.2. Assessing the Efficiency of Time Utilization

In this study, we conducted tests to measure the time consumption of the main modules processing each frame, with tracking time closely tied to computer performance and the quantity of extractions. In the tracking thread of this paper, the average processing time per frame is 135 milliseconds. As depicted in Table 4, a comparative time analysis was performed to assess the average processing time of DS-SLAM and our developed system in terms of target segmentation and feature extraction in real-world and fr3/w/xyz scenes.

Table 4 illustrates the average time consumption for processing each frame between DS-SLAM and IPL-SLAM. It is evident from Table 4 that in the feature extraction phase, IPL-SLAM takes longer to extract features per frame due to the addition of line feature extraction compared to DS-SLAM, which exclusively extracts point features. Concerning the extraction of object region information in each frame’s image segmentation, YOLOv8 demonstrates finer segmentation but requires more time compared to SegNet, with an average time of 126.6 milliseconds.

In addition to reporting the average runtime, we have analyzed the time complexity of the main modules. For each RGB-D frame with $N_{p}$ pixels and $N_{f}$ extracted features, the ORB feature extraction operates in $O(N_{p})$, while the LSD line detector scales approximately with $O(N_{p}\log N_{p})$ due to the multi-scale edge refinement. The YOLOv8-based instance segmentation runs in $O(N_{p})$ with a constant-depth convolutional backbone. The overall pipeline complexity per frame can therefore be approximated as $O(N_{p}\log N_{p}+N_{f})$. Compared to ORB-SLAM2, which primarily operates in $O(N_{f})$, the additional segmentation and line feature modules explain the higher runtime of IPL-SLAM. For reference, DynaSLAM incurs $O(N_{p})$ Mask R-CNN inference per frame, while YOLO-SLAM maintains $O(N_{p})$ but with less post-processing due to bounding-box segmentation. We have added this complexity analysis alongside Table 4 to contextualize the reported average time of 126.6 ms per frame for finer segmentation.

At the same time, in view of the time-consuming modules such as contour similarity analysis and line feature extraction in the dynamic region filtering algorithm for consecutive image sequences, and considering the real-time requirements, this paper deals with the operations of line feature extraction and contour similarity comparison through separate threads. Moreover, in order to make full use of the multi-core characteristics of the CPU, the IPL-SLAM system uses CPU instruction -set optimization, especially SIMD (Single Instruction Multiple Data) technology, to significantly improve computational efficiency in engineering applications. For example, the calculation of Hu moments involves a large number of mathematical operations, such as squaring, multiplication, and summation. These operations can be vectorized through SIMD instruction sets (such as AVX or SSE), thereby significantly increasing computational speed. When dealing with image frames under consecutive time sequences within a sliding window, SIMD instruction sets can be utilized to process the data of multiple contours in parallel. The pixel values of multiple contours are loaded into SIMD registers, and squaring and summation operations are performed simultaneously, which reduces the number of loops and increases computational efficiency. In addition, during the process of line feature extraction and line feature descriptor matching, a large number of geometric calculations and logical judgments are involved. IPL-SLAM parallelly processes multiple matching pairs through SIMD instruction sets, thereby increasing the verification speed.

Additionally, regarding the time-consuming issue of image segmentation, we will subsequently employ methods such as model quantization and inference acceleration to speed up the image segmentation process. Meanwhile, we will also seek more lightweight models to replace the existing ones while ensuring the segmentation accuracy.

### 4.3. Experiments in Real-World Environments

**Figure 14:**
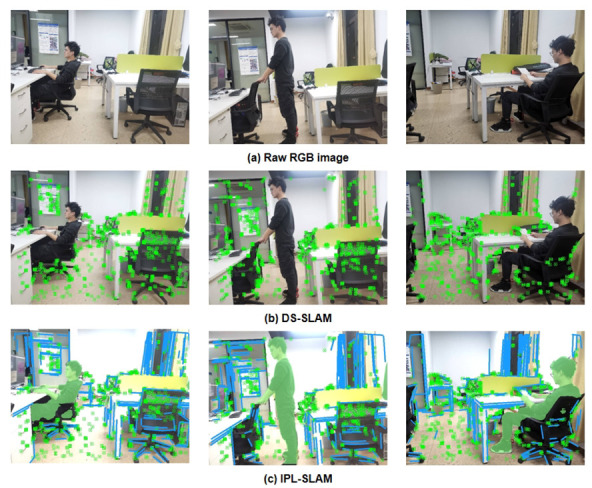
Comparison of extracted static feature sequences: (**a**) shows the collected raw image data; (**b**) presents the static points features extracted by DS-SLAM; (**c**) shows the static point and line features extracted by IPL-SLAM.

To further assess the performance of our developed system in real-world scenarios, we captured images of dynamic indoor scenes using a Kinect2 camera and processed the collected image data in accordance with the TUM dataset format. The comparative effectiveness of static feature extraction between DS-SLAM and IPL-SLAM in real scenarios is demonstrated in Figure 14. Figure 14a depicts a scenario in which a person stands up from a chair beside the table on the left, walks towards the table on the right, and then sits down. The comparison indicates that IPL-SLAM surpasses DS-SLAM in eliminating dynamic points and dynamic lines, as well as in the effective utilization of line features within the environment.

**Figure 15:**
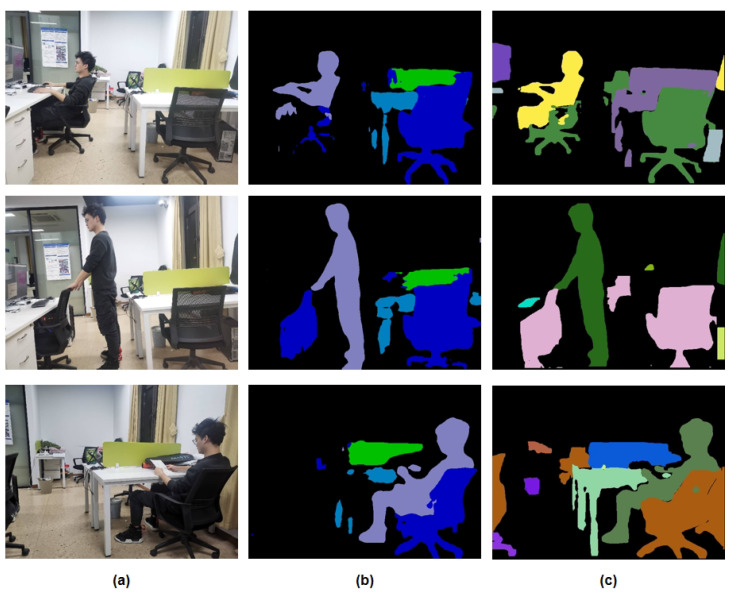
(**a**) Raw RGB image; (**b**) DS-SLAM segmentation; (**c**) IPL-SLAM segmentation.

At the same time, as illustrated in Figure 15, IPL-SLAM, developed based on the YOLOv8 instance segmentation network, exhibits superior segmentation performance compared to DS-SLAM.

### 4.4. Point Cloud Map

In this section, we generate a static 3D point cloud map using the point cloud construction module. Firstly, the module combines the masks and depth maps of the previously generated dynamic objects to create a point cloud map that does not contain dynamic objects. Secondly, the point cloud is filtered using a downsampling algorithm, after which the local point clouds are merged into a global point cloud to generate the final global static point cloud map.

**Figure 16:**
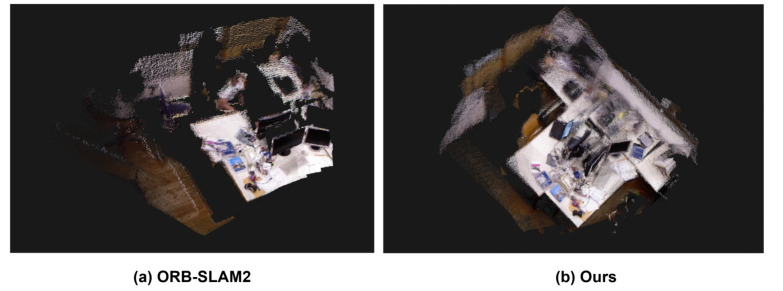
Comparison of point cloud maps constructed by ORB-SLAM2 and our system.

The experimental results are presented in Figure 16. Figure 16a shows the point cloud map generated by the ORB-SLAM2 algorithm, which includes duplicate images of a person in the point cloud because dynamic objects were not filtered out. In contrast, Figure 16b shows the point cloud map generated after applying our dynamic object filtering algorithm.

As depicted in the figure above, ORB-SLAM2 is unable to effectively filter out dynamic human entities in dynamic environments. Consequently, erroneous feature point matches at the periphery of human figures lead to inaccurate pose estimation, which in turn gives rise to the “ghosting” phenomenon of the point cloud near humans. In contrast, the algorithm proposed in this paper incorporates a dynamic object filtering module, which classifies humans as dynamic objects and removes them. As a result, the estimated pose near the original human locations is more accurate, thereby producing a clearer point cloud map.

## 5. Conclusions

In this paper, a biologically-inspired semantic RGB-D SLAM system—IPL-SLAM—has been presented for real-time localization in dynamic environments. The system integrates YOLOv8-based instance segmentation with geometric feature extraction to improve the robustness and accuracy of pose estimation. Dynamic regions are identified using pixel-level semantic masks combined with epipolar and angular consistency checks on point and line features. An adaptive-weight error function is introduced to optimize camera pose by jointly leveraging static points and lines. A static semantic point cloud map is then constructed by combining the optimized pose with depth information, enabling higher-level scene perception for autonomous agents.

Although YOLOv8 is capable of providing polygonal boundaries and, with specific configurations, keypoint locations for certain object classes, we adopt a custom post-processing algorithm to extract line segments and keypoints from the binary masks. This choice is motivated by several factors: (1) the polygonal outputs of YOLOv8 are optimized for object detection tasks and may exhibit irregularities at small-scale or partially occluded objects, whereas our method enforces geometric consistency suitable for SLAM back-ends; (2) extracting line and point features through the same LSD/ORB pipeline ensures alignment between semantic masks and geometric constraints, reducing potential drift between segmentation and feature tracking; (3) YOLOv8’s keypoint detection is class-specific (e.g., human pose estimation), while the proposed approach provides a class-agnostic mechanism applicable to arbitrary dynamic objects. This integration yields more stable point–line fusion and consistent dynamic feature suppression in the SLAM pipeline.

The proposed system has been evaluated on both the TUM RGB-D dataset and real-world scenarios. Experimental results demonstrate that IPL-SLAM significantly improves localization accuracy and robustness compared to DS-SLAM and other dynamic SLAM methods, especially in highly dynamic indoor scenes. Even under low-dynamic conditions, further improvements over baseline systems are observed, validating the effectiveness and generalizability of the approach.

Despite these promising results, several limitations remain. The computational cost introduced by instance segmentation and line feature matching affects real-time performance. To further improve computational efficiency, future work will explore model pruning and quantization for the segmentation network, GPU-accelerated line feature extraction, and parallel processing strategies. Additionally, integrating lightweight instance segmentation models or leveraging hardware-friendly neural network architectures could help achieve real-time performance on embedded platforms. In texture-scarce environments, the weighting scheme for dynamic suppression may be less effective; hence, improved strategies will be explored. We also plan to store the categories of objects previously identified with YOLOv8 to accelerate dynamic object recognition. In scenes with multiple instances of the same category, false positives may occur when new objects appear. Future versions will incorporate instance-level tracking to reduce such issues.

Finally, while our evaluation focuses on indoor environments, IPL-SLAM has the potential to be extended to outdoor or large-scale scenarios. This extension will require addressing challenges such as varying illumination, wide-baseline viewpoints, long-range depth perception, and efficient loop closure over extended trajectories. We plan to explore these directions in future work, potentially integrating event-based cameras and lightweight semantic networks to enhance applicability in high-speed or resource-constrained settings.

## Figures and Tables

**Table 1:** Comparison of camera pose estimation between DS-SLAM and IPL-SLAM according to the absolute trajectory error (ATE [m]). Bold entries denote the data that produced a more significant effect in the experiment.

	DS-SLAM				IPL-SLAM			
Seq.	RMSE	Mean	Median	STD	RMSE	Mean	Median	STD
fr3/w/xyz	0.0288	0.0206	0.0166	0.0201	**0.0129**	**0.0112**	**0.0105**	**0.0068**
fr3/w/rpy	0.4752	0.4051	0.2856	0.2484	**0.0827**	**0.0592**	**0.0526**	**0.0415**
fr3/w/half	**0.0283**	**0.0242**	**0.0209**	**0.0147**	0.0315	0.0269	0.0226	0.0164
fr3/w/static	**0.0076**	**0.0069**	**0.0063**	**0.0036**	0.0113	0.0104	0.0098	0.0043
fr3/s/rpy	0.0213	0.0155	0.0118	0.0146	**0.0198**	**0.0149**	**0.0109**	**0.0122**

**Table 2:** Comparisons of absolute pose error among different systems (ATE [m]). Bold entries denote the data that produced a more significant effect in the experiment.

Seq.	ORB-SLAM2	Dyna-SLAM	CFP-SLAM	YOLO-SLAM	PLD-SLAM	DPL-SLAM	IPL-SLAM
fr3/w/xyz	0.5359	0.0158	0.0141	0.0194	0.0144	0.0627	**0.0129**
fr3/w/rpy	0.7408	0.0402	0.0368	0.0933	0.2212	**0.0310**	0.0827
fr3/w/half	0.3962	0.0274	**0.0237**	0.0268	0.0261	0.0242	0.0315
fr3/w/static	0.3540	0.0080	0.0066	0.0094	**0.0065**	0.0068	0.0113
fr3/s/rpy	0.0214	0.0302	0.0253	–	0.0222	–	**0.0198**

**Table 3:** Ablation experiments of camera pose estimation based on the ATE (m) metric. (Bold entries denote the data that produced a more significant effect in the experiment).

Seq.	YOLOv8	YOLOv8 + LSD	YOLOv8 + Motion Consistency Checks	YOLOv8 + LSD + Adaptive Weights
fr3/w/xyz	0.012976	0.012945	0.012923	**0.012909**
fr3/w/rpy	0.082932	0.082801	0.082795	**0.082748**
fr3/w/half	0.031625	**0.031523**	0.031567	0.031549
fr3/w/static	0.011356	0.011332	0.011341	**0.011324**

**Table 4:** Comparison of time efficiency between DS-SLAM and IPL-SLAM (unit: ms).

Seq.	ORB Feature Extraction (DS-SLAM)	SegNet (DS-SLAM)	ORB + LSD Feature Extraction (IPL-SLAM)	YOLOv8 (IPL-SLAM)
fr3/w/xyz	15.65	75.25	56.35	125.18
Real-world scene	18.23	78.36	58.56	127.35

## Data Availability

The data presented in this study are available on request from the corresponding author. The data are not publicly available due to institutional policy and storage limitations.
